# A Developmental Model for Predicting Sport Participation among Female Korean College Students

**DOI:** 10.3390/ijerph17145010

**Published:** 2020-07-12

**Authors:** Sung-Un Park, Chung Gun Lee, Dong-Kyu Kim, Jong-Hwa Park, Deok-Jin Jang

**Affiliations:** 1 Department of Sport & Leisure Studies, College of Arts & Physical Education, Shingyeong University, Hwaseong 18274, Korea; psu@sgu.ac.kr; 2 Department of Physical Education, College of Education, Seoul National University, Seoul 08826, Korea; 3 Department of Sport Management, Graduate School of Technology Management, Kyung Hee University, Yongin 17104, Korea; 4 Major of Taekwondo, College of Sport Science, Dankook University, Cheonan 31116, Korea; parkjh329@dankook.ac.kr; 5 Department of Coaching, College of Physical Education, Kyung Hee University, Yongin 17104, Korea; tmzlqn@khu.ac.kr

**Keywords:** competency, relatedness, autonomy, theory of planned behavior, female Korean college students

## Abstract

Although participating in regular physical activity has many benefits, female Korean college students tend to have much lower participation rates than their male counterparts. An effective means of increasing physical activity among female college students is sport participation. The purpose of this study is to incorporate three types of psychological needs from self-determination theory as precursor background variables into the theory of planned behavior to predict sport participation among female Korean college students. Our dataset consisted of 494 female undergraduate students attending Kyung Hee University in South Korea. Using structural equation modeling, the direct and indirect effects of attitude, subjective norm, perceived behavioral control, and psychological needs satisfaction such as competency, relatedness, and autonomy were examined. Although attitude towards and perceived behavioral control over sport participation were significantly associated with intention in all three models, subjective norm was not significantly associated with intention in any model. Satisfaction of the psychological needs for competency, relatedness, and autonomy had positive indirect effects on sport participation. This study underscores the importance of addressing the satisfaction of these three basic psychological needs when designing future sport promotion interventions for female college students.

## 1. Introduction

Although participating in regular physical activity has many benefits [1,2,3,4], only 20.8% of college students in South Korea engage in the recommended level of exercise, which is much lower than the rate of 52% among US college students who engage in moderate to vigorous activity [5,6]. Moreover, female college students in South Korea have been shown to have much lower rates of physical activity (13.6%) than their male counterparts (34.2%) [6]. Given the health benefits of exercise, more efforts should be made to encourage it, especially among Korean female college students, because lifestyle behaviors established during college years often remain unchanged throughout an individual’s life [7].

An effective way of increasing physical activity among female college students is sport participation. Participation in sport activities includes such enjoyable aspects as playful competition, social interaction, goal achievement, and overcoming personal challenges. Various studies have identified useful reasons for participating in sport activities, such as improved fitness, social interaction, and enjoyment [8,9]. Since participation in sport activities invariably involves exercise, it is important to develop efficient sport promotion programs for female college students. Such programs should be based on sound theoretical frameworks to better ensure desirable outcomes [10]. Thus, research is needed to determine the most effective theoretical framework [11].

**Figure 1:**
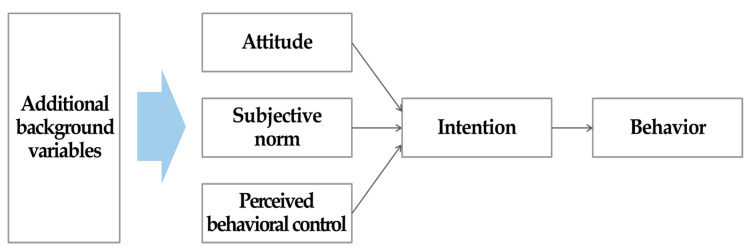
Theory of planned behavior with additional background variables as precursor variables (Hennessy et al., 2010) [18].

One of the most frequently applied frameworks for predicting health-related behavior is the theory of planned behavior (TPB) [12]. The TPB assumes that the most proximal determinant of a given behavior is the intention to perform that behavior; this is strongly affected by the subjective norm (social approval/disapproval) and attitude (positive/negative evaluation) related to the behavior and the perceived behavioral control (ease/difficulty) over its performance [13]. Following Ajzen and Albarracin’s suggestion [14] that the TPB can accommodate additional predictors, we drew on this theory to identify additional background variables that might improve its predictive power. A number of studies on physical activity-related behaviors have incorporated an extensive array of predictors within the context of the TPB [15,16,17]. Although their results support the notion that the inclusion of additional factors within the TPB can improve its predictive power, most of these studies have a major limitation. Specifically, the use of multiple regression models has led most prior research to treat additional predictors as control rather than precursor variables. A recent study found substantial differences in the results of studies treating additional variables as control variables versus precursor variables [18]. Hennessy et al. [18] also suggested that the effects of all additional predictors incorporated into the TPB should be assumed to be mediated by the core TPB constructs because the additional predictors occur prior to those constructs (Figure 1).

According to the self-determination theory (SDT), individuals struggle to fulfil the basic psychological needs of competence, autonomy, and relatedness. SDT suggests that these three universal psychological needs must be satisfied for individuals to maintain optimum performance and wellbeing. SDT also states that as these basic psychological needs are complementary, for optimum psychological functioning and the maintenance of a particular behavior, all three must be supported by the social context [19]. Integrating the concept of psychological needs satisfaction into theories of motivation such as the TPB is desirable because it allows us to explore the relative contribution of these global motives on situation-level predictors of behavior (e.g., attitude, subjective norms, and perceived behavioral control) in various contexts [20]. However, to our knowledge, only one study has incorporated psychological needs satisfaction as a precursor background variable into the TPB to predict physical activity behavior, and it did not distinguish among different types of psychological needs [21].

Therefore, the current study used structural equation modelling to incorporate three types of psychological needs from SDT—competence, autonomy, and relatedness—as precursor background variables into the TPB to determine the differences among the effects of these needs on sport participation in female college students and predict sport participation among the respondents. The following research questions were addressed: (1) how does satisfaction of each of the three identified psychological needs indirectly affect sport participation through the three components of the TPB (i.e., attitude, subjective norms, and perceived behavioral control) and intention? (2) How do the effects of each type of psychological need differ?

## 2. Methodology

### 2.1. Data

This study received approval from the Kyung Hee University Global Campus-designated institutional bioeth-ics committee (KHGIRB-19-327). From March–May 2020, a convenience sample of female undergraduate students of Kyung Hee University (Seoul and Global Campus) in South Korea was asked to complete a self-administered questionnaire. Professors agreed to conduct the survey during their classes. Students were informed of the main purpose of this study. Although structural equation modeling does not have an exact standard for sample size, the number of participants should be at least 15 times the number of observed variables to be measured, and it is generally recommended to have a sample size of 200 or more [22]. In total, this study analyzed 494/500 sets of data (98.8%; six unre-liable responses were discarded), which met the recommended sample size and satisfied the minimum number of participants. The mean score of sport participation was 64 min per week. The mean age was 20.36 years. Most were freshmen (35.02%), and the majority were living with their parents (71.05%). More than half of the participants had middle household incomes (49.19%). Table 1 presents the descriptive statistics of the participants.

### 2.2. Measures

Intention to participate in sports was measured with four questions, such as ‘I intend to participate in sports in the next month’. These items were rated on a 5-point Likert scale ranging from very unlikely to very likely. Participants reported their attitudes towards participating in sports using semantic differential measures of ‘good or bad’, ‘wise or unwise’, ‘desirable or undesirable’, and ‘beneficial or harmful’. These items were rated on a 5-point Likert scale. Subjective norm was measured with four questions, including ‘most people who are important to me think I should participate in sports in the next month’. These items were rated on a 5-point scale ranging from very unlikely to very likely. To assess perceived behavioral control, participants answered eight items asking how easy they thought it would be for them to participate in sports within the next month. These items were rated on a 5-point Likert scale. Validated measures of basic psychological need satisfaction in the context of sport participation were used to assess the degree to which participants felt their needs of autonomy, competence, and relatedness were satisfied [19]. Items measuring satisfaction of the psychological need for autonomy (e.g., ‘I feel like I am free to decide for myself what I will do when I participate in sports’), relatedness (e.g., ‘I consider the people I regularly interact with during sport participation to be my friends’), and competence (e.g., ‘I have been able to learn interesting new sport skills recently’) were measured using 21 items. These items were scored on a 5-point Likert scale ranging from not true at all to very true. Participants were explicitly instructed on rating the scales according to how they felt during their sport participation. Sport participation was measured by asking participants to report the total time (minutes) spent participating in sports during the past week. Socio-demographic characteristics of the participants such as age (years), college year (freshman, sophomore, junior, or senior), residence type (living with parents, living apart from parents (off-campus), living apart from parents (on-campus), and others), and household income (low, middle, and high) were also assessed.

### 2.3. Statistical Analysis

We examined the direct and indirect influences of the following variables on sport participation among Korean female college students: perceived behavioral control, subjective norms, attitude, and precursor background variables (i.e., the basic psychological needs of autonomy, competence, and relatedness). Mplus Version 7 (Muthen & Muthen, Los Angeles, CA, USA) was used to perform the structural equation modelling analysis [23]. All variables were continuous, and this study used confirmatory factor analysis to examine a measurement model that contains five correlated latent variables (i.e., satisfaction of each of the three types of psychological need, subjective norms, attitude, perceived behavioral control, and intention). The Sobel method [24] was used to examine indirect effects. If the sample size is small, the Sobel test may incorrectly assume normality of the indirect effect [24]. Therefore, bootstrapped standard errors were used for the test of indirect effects [25]. The order in which the variables were entered into the model was based on the hypothesized theoretical framework. We used three model fit indices to assess goodness of fit (root mean square error of approximation (RMSEA), chi square, and comparative fit index (CFI)).

## 3. Results

The standardized paths from each latent variable to its respective items were examined. We allowed correlations between all latent variables in the model specification. In our three measurement models, although the chi-square values were all significant (*ps* < 0.05), the RMSEAs ranged from 0.083 to 0.094 and the CFIs ranged from 0.867 to 0.900, indicating that our measurement models represented the data accurately [26,27,28].

**Figure 2:**
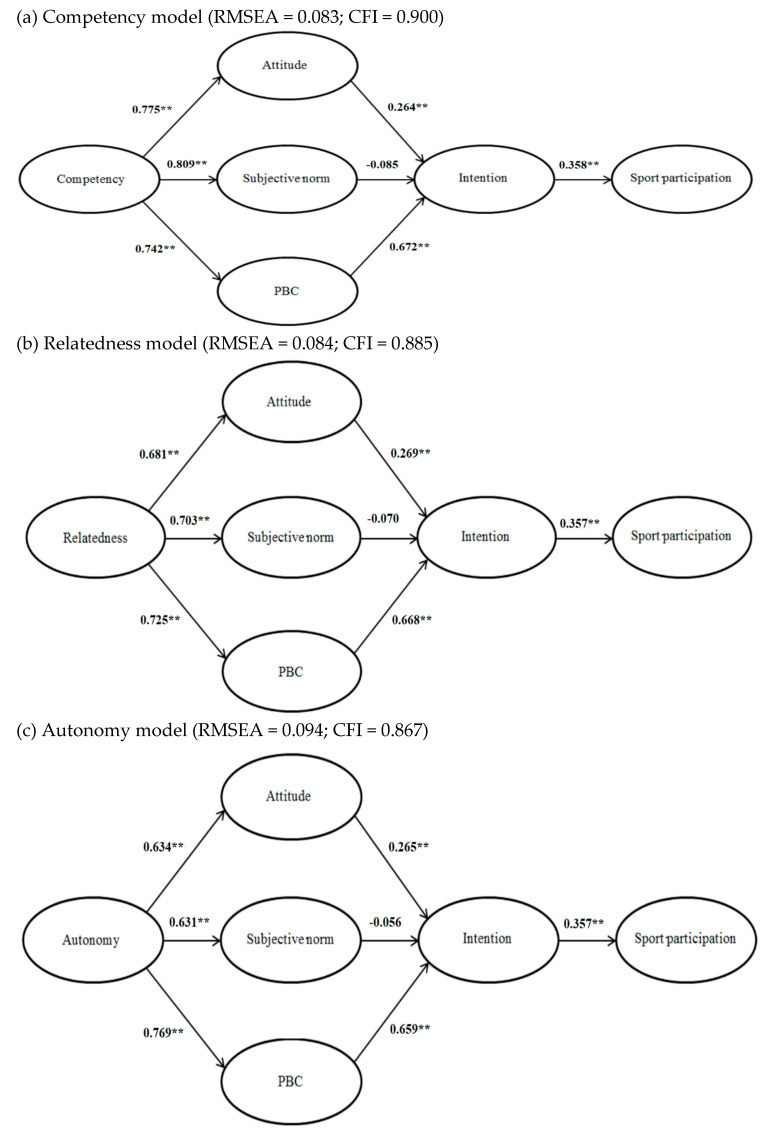
The structural models. Note: ** *p* < 0.01; PBC = perceived behavioral control. All path coefficients were standardized. (**a**) Competency model (RMSEA = 0.083; CFI = 0.900). (**b**) Relatedness model (RMSEA = 0.084; CFI = 0.885). (**c**) Autonomy model (RMSEA = 0.094; CFI = 0.867).

Figure 2 shows the path coefficients of the three structural equation models. Our study observed a similar pattern of results across the three models. Intention significantly predicted sport participation in all three models, and attitude and perceived behavioral control were significantly associated with intention in all three models. However, subjective norm was not significantly associated with intention in any model.

Satisfaction of all the three psychological needs for competency, relatedness, and autonomy was positively associated with attitude, subjective norm, and perceived behavioral control.

The indirect effects of additional background variables on sport participation through the three components of the TPB and intention were also examined (Table 2). Satisfaction of all the three psychological needs had positive indirect effects on sport participation through attitude and perceived behavioral control. Subjective norm did not mediate the impact of satisfaction of psychological needs on sport participation

## 4. Discussion

The present study incorporated psychological needs satisfaction as a precursor background variable in the TPB to predict sport participation among female Korean college students. According to Noar and Zimmerman [29], the synthesis or integration of different theories can lead researchers to capitalize on the strengths of each theory, thereby enhancing the effectiveness of the development of theory-based interventions or programs. This study attempted to reinforce previous studies [15,16,17,21] by (1) incorporating psychological needs satisfaction as a precursor background variable (rather than a control variable) into the TPB to predict sport participation; (2) examining the differences among the effects of each type of psychological need (i.e., competence, autonomy, and relatedness) on sport participation; (3) using a formal significance test of indirect effects to investigate whether the additional background variables indirectly affect sport participation through the three main constructs of the TPB and intention.

Although attitude towards and perceived behavioral control over sport participation were both significantly associated with intention in all three models, subjective norms were not significantly associated with intention in any model. This result is consistent with a recent review of the application of the TPB to health-related behaviors which found that attitude was the most powerful predictor of intention, while the magnitude of the effect of perceived behavioral control on intention was similar to that of attitude [30]. The insignificant effect of subjective norm on intention may be due to the traditional patriarchal system in South Korea. Many societies educate women with the goal of polarizing femininity and masculinity, and this cultural framework can label women who participate in sport as deviant or maladjusted [31]. Such an atmosphere may threaten women’s identities as feminine [32] and thus limit female Korean college students’ opportunities for sport participation and desire to engage in such activity [33]. As seen in previous research, the rate of regular physical activity in female college students is much lower than in male college students in South Korea [6].

The results of this study showed that satisfaction of the psychological needs for competency, relatedness, and autonomy had positive indirect effects on sport participation through attitude, perceived behavioral control, and intention. According to Hagger et al. [21], physical-activity-related behavior does not occur spontaneously. Instead, people engage in physical activity because of their need for satisfaction of psychological needs by carefully considering the activities in which they are involved, such as sports that can generate autonomous goals and values, thereby helping them make plans to exercise. Our results provide independent support for the relative contribution of global motives on situation-level predictors of behavior, such as attitude and perceived behavioral control [34,35,36]. The integration of the TPB into SDT provides insights into the mechanisms through which global psychological needs satisfaction affects the decision to participate in sport at the situational level. The target population of this study was female Korean college students, a group that tends to be less likely to participate in sport activities [37]. Providing a variety of opportunities for women to develop interest in sports and creating a social environment within which women are comfortable living a physically active lifestyle are likely to be effective in promoting sport participation among women around the world [38].

The results of this study are subject to several limitations. First, all data were self-reported, which might have resulted in respondent, recall, and/or interviewer biases. Traditional Asian cultural values, such as sexual conservativism and face-saving behavior, might have led the respondents to under-report their perceptions and behaviors. Second, due to the cross-sectional design of this study, causal relationships among the variables could not be determined. Some caution is thus needed when interpreting the findings. Third, the results may not be generalizable to other populations because we focused exclusively on female Korean college students.

## 5. Conclusions

Despite these limitations, the findings contribute to the literature by providing valuable information on the need to consider environmental variables when predicting sport participation in female college students. Our findings are important because sport participation among South Korean women has been largely ignored over the last two decades. Irrespective of how intensely female Korean college students believe that people important to them want them to participate in sports, larger-scale social norms, such as the traditional patriarchal system, may lead them to avoid sport activity. Our results also suggest that creating autonomy, developing a sense of competence, and providing a supportive social environment are important in encouraging sport participation among this cohort. This study underscores the importance of addressing the satisfaction of psychological needs when designing sports promotion interventions for this under-represented group.

## Figures and Tables

**Table 1:** Socio-demographic characteristics of participants (*n* = 494).

Characteristics	Mean	SD
Sport participation (minute/week)	64.18	122.94
Age (years)	20.36	1.83
	*n*	%
18	103	20.9
19	81	16.4
20	62	12.6
21	143	28.9
22	27	5.5
23	58	11.7
24	11	2.2
25	5	1.0
26	3	0.6
27	1	0.2
College year		
Freshman	173	35.02
Sophomore	77	15.58
Junior	163	33
Senior	81	16.4
Residence type		
Living with parents	351	71.05
Living apart from parents (off-campus)	83	16.81
Living apart from parents (on-campus)	51	10.32
Others	9	1.82
Household income		
Low	166	33.61
Middle	243	49.19
High	85	17.2

Note: SD = standard deviation.

**Table 2:** The indirect effects of self-determination theory constructs on sport participation.

Indirect Effects				Coefficients
Competency	Attitude	Intention	Sport participation	0.073 **
	Subjective norm	Intention	Sport participation	−0.025
	Perceived behavioral control	Intention	Sport participation	0.179 **
Relatedness	Attitude	Intention	Sport participation	0.065 **
	Subjective norm	Intention	Sport participation	−0.017
	Perceived behavioral control	Intention	Sport participation	0.173 **
Autonomy	Attitude	Intention	Sport participation	0.060 **
	Subjective norm	Intention	Sport participation	−0.013
	Perceived behavioral control	Intention	Sport participation	0.181 **

Note: ** *p* < 0.01.
